# Measuring the impact of virtualization and containerization on the environment when using GPUs for processing the AI models

**DOI:** 10.3389/fdata.2026.1838191

**Published:** 2026-06-16

**Authors:** Safaa Hriez, Mohammad Haikal

**Affiliations:** 1 Cyber Security Department, Al Hussein Technical University, Amman, Jordan; 2 SparklyMinds, Amman, Jordan

**Keywords:** AI workloads, artificial intelligence (AI), computer vision, containerization, energy consumption, energy efficiency, environmental impact, Graphics Processing Unit (GPU)

## Abstract

**Introduction:**

The rapid growth of artificial intelligence (AI) has significantly increased computing demand, intensifying the operational strain on the computing environment. While virtualization and containerization are established technologies for resource optimization, their comparative energy efficiency and environmental impact, particularly under GPU-accelerated AI workloads, are not well-quantified.

**Methods:**

This study evaluates the energy consumption and environmental impact of virtualization and containerization technologies when using Graphics Processing Units (GPUs) for AI model execution. Employing a computer vision benchmark, the performance, GPU resource utilization, and power consumption were measured. The experiment involved training a DenseNet-121 model on the MNIST dataset within a VirtualBox virtual machine and a Docker container environment.

**Results:**

The analysis indicates that containerization consistently surpasses virtualization in energy efficiency. Specifically, Docker container configuration demonstrated an approximately 21.6% reduction in total energy consumption, and a corresponding reduction in carbon dioxide (CO_2_) emissions compared to a VirtualBox virtual machine. Furthermore, containerization exhibited lower average and peak GPU utilization and power consumption.

**Discussion:**

These findings demonstrate that containerization offers a more energy-efficient and environmentally sustainable approach than VirtualBox virtualization for the specific GPU-enabled AI workload evaluated in this study. Statistical significance testing indicates that the observed performance differentials are significant, supporting the validity of the results within the experimental scope of this work.

**Conclusion:**

Implementing containerization in this experimental setup may reduce energy consumption and environmental impact without compromising computational performance. Future studies should extend these analyses to larger neural network models, diverse AI workloads, and heterogeneous GPU platforms to enhance the generalizability of these findings beyond the current single-system experimental configuration.

## 1 Introduction

The growing threat of climate change poses an escalating risk to life on Earth, disproportionately impacting vulnerable ecosystems and marginalized societies. Extreme weather events, resource scarcity, and food insecurity are increasingly undermining livelihoods and exacerbating global inequality (United Nations, 2023). Carbon dioxide (*CO*_2_) remains a primary driver of global warming due to its heat-trapping properties. As global emissions from fuel combustion continue to rise, high-energy industries such as data centers significantly contribute to *CO*_2_ output (International Energy Agency, 2025).

Anthropogenic *CO*_2_ emissions arise mainly from fossil fuel combustion in transportation, heating, and industrial sectors. A rapidly growing contributor is digital infrastructure, including devices, networks, and data centers supporting cloud computing, artificial intelligence (AI), and digital services. This ecosystem accounts for approximately 1% of global greenhouse gas emissions, with projections indicating further increases (London School of Economics and Political Science, 2025). Therefore, reducing emissions from the IT sector has become an urgent environmental priority, requiring sustainable technological development (Reuters, 2025).

Cloud computing is now a dominant paradigm in the IT industry, widely adopted for scalable and efficient service delivery. However, its rapid growth has significantly increased data center energy consumption. Recent studies show that energy demand from data centers continues to rise alongside AI and compute-intensive workloads, leading to higher carbon emissions (International Energy Agency, 2025; Masanet et al., 2020). As a result, improving energy efficiency at the software level has become essential, since software design directly influences resource utilization and power consumption. Prior research has highlighted that inefficient software can offset hardware-level optimizations, increasing overall energy usage. This has motivated research into energy-aware scheduling and resource optimization techniques for sustainable data centers (Kumar and Pandey, 2016). Virtualization and containerization have also been widely studied for improving resource utilization and reducing infrastructure overhead, with container technologies such as Docker gaining increasing attention due to their lightweight design and efficiency advantages (Pahl et al., 2017).

**Figure 1:**
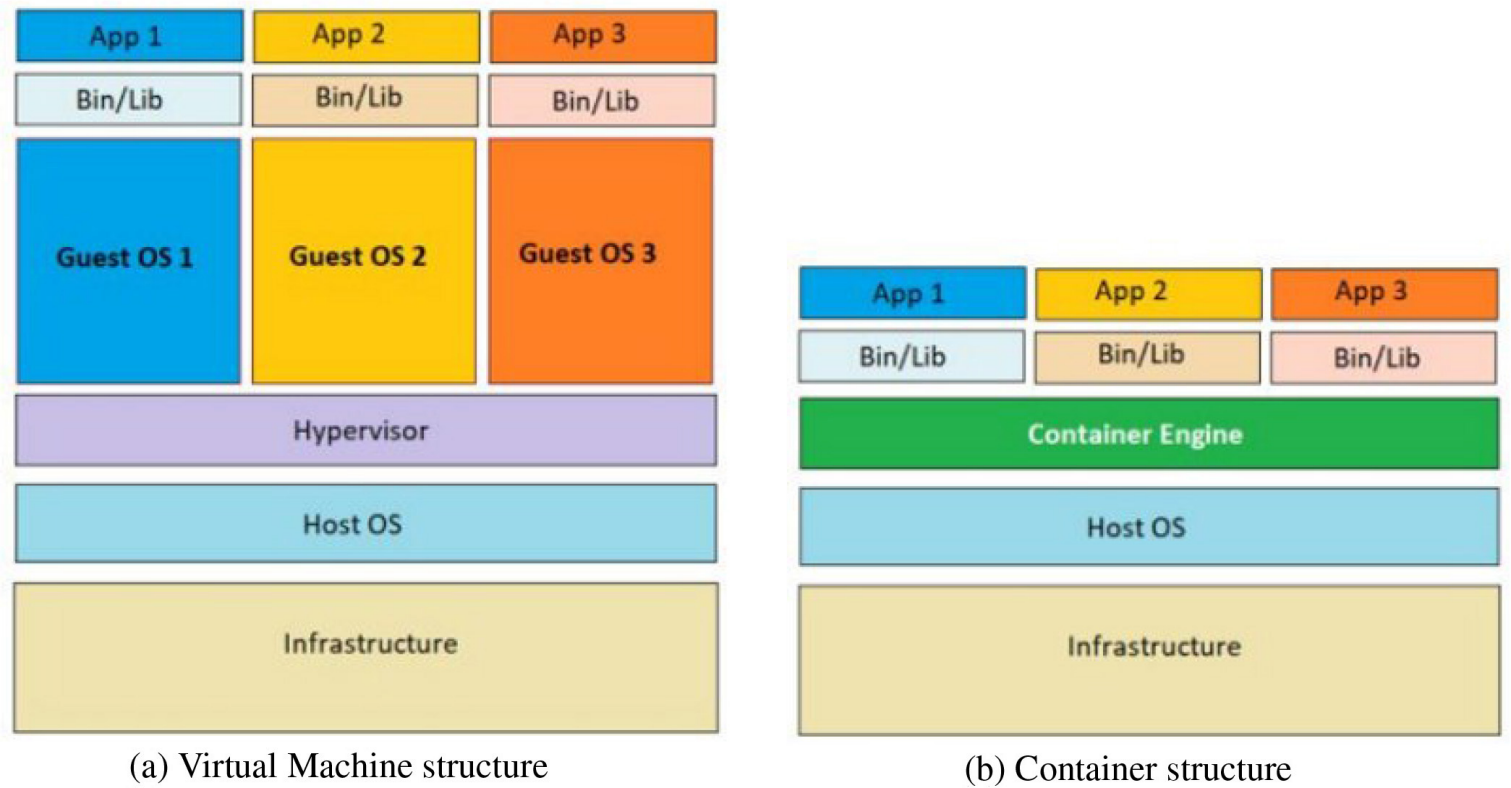
Comparison between virtual machine and container architectures. **(a)** Virtual machine structure (Cyril, 2021). **(b)** Container structure (Cyril, 2021).

Virtualization improves hardware utilization by allowing multiple workloads to run on a single physical machine through a hypervisor layer, which manages virtual machines (VMs), as shown in Figure 1a. This enables concurrent execution of multiple operating systems and applications, improving resource efficiency and reducing idle capacity. Consequently, virtualization has been widely adopted in data centers to enhance scalability and reduce energy consumption (Anusooya and Vijayakumar, 2021).

A container is a lightweight virtualization mechanism that operates at the operating system level, sharing the host kernel while isolating applications. Unlike traditional virtualization, containers avoid the overhead of separate guest operating systems, resulting in improved efficiency and faster execution. Container engines manage resource allocation, isolation, and scalability, enabling efficient deployment of applications, as illustrated in Figure 1b. Prior studies have shown that containerization improves resource utilization and reduces energy consumption in cloud environments compared to virtual machines (Pahl et al., 2017; Anusooya and Vijayakumar, 2021).

This study investigates the environmental effects of virtualization and containerization when using Graphics Processing Units (GPUs) for AI model training. The analysis is conducted through a computer vision task, providing an empirical evaluation of performance and efficiency. The principal contributions of this study are as follows:

1. Comparative Assessment of Virtualization and Containerization in AI Workloads: This study provides a comprehensive comparison between VirtualBox VMs and Docker container technologies. The analysis focuses on GPU utilization metrics, power consumption profiles, and the resulting environmental impact during the training phase of AI model. The results demonstrate that Docker containers offer superior energy efficiency, reduced resource overhead, and more consistent performance stability relative to VirtualBox VMs.
2. Quantitative Assessment of Energy Consumption and Environmental Impact: Through rigorous experimentation, this study measures the total energy usage consumption and derives estimated *CO*_2_ emissions for both VirtualBox VM and Docker container configurations. The findings reveal that Docker containers facilitate a reduction in energy consumption of approximately 21.6% and a corresponding 22% decrease in *CO*_2_ emissions compared to VMs. These findings provide concrete, data-driven insights for developing more sustainable AI computing infrastructure.
3. Statistical Validation of Observed Performance Variances: The study employs independent samples t-tests to statistically validate the differences in power consumption and GPU utilization between VirtualBox VMs and Docker containers. The results provide robust empirical evidence that the efficiency gains observed in Docker are highly statistically significant (p < 0.001).
4. Practical Recommendations for AI Workload Deployment: Based on the experimental outcomes of this study, we provide recommendations for a controlled GPU-based AI execution setting. The results suggest that, within the tested configuration, containerization may offer improved energy efficiency compared to VirtualBox virtualization for the evaluated AI workload. This approach can help reduce measured power consumption and improve resource utilization under similar experimental conditions, potentially leading to lower environmental impact in comparable setups.
5. Advancement of Sustainable AI and Green Computing Practices: By quantifying the environmental differences between containerization and VirtualBox virtualization within a controlled experimental GPU-based AI workload, this work contributes empirical observations relevant to green computing discussions at the system level. The findings provide an empirical basis for infrastructure comparisons in similar experimental configurations, showing differences in measured energy usage and estimated carbon emissions. These results are limited to the tested setup; however, they may serve as a preliminary reference point for future studies aimed at broader and more generalized evaluations of energy-related trade-offs in virtualization technologies.

The structure of this paper proceeds as follows. The Literature Review section synthesizes key findings and methodological approaches from existing studies relevant to the topic. The Methodology section details the experimental design, data collection procedures, and analytical methods employed in the study. The Results and Discussion section presents the experimental outcomes and provides a critical interpretation of their scientific and practical significance. The paper concludes by summarizing the principal insights, acknowledging limitations, and proposing directions for future research aimed at advancing sustainable computing practices.

## 2 Literature review

Previous studies have extensively investigated the performance and energy consumption characteristics of virtualization technologies, with a primary focus on comparisons between hypervisor-based virtual machines and container-based solutions. Early investigations consistently report that containers impose significantly lower computational overhead than traditional VMs. This efficiency advantage translates to measurable improvements in application performance and reduction in power consumption, particularly for CPU-, memory-, and I/O-intensive workloads.

Morabito (2015a) and Tadesse et al. (2018) demonstrated that containers generally exhibit lower power consumption than virtual machines under idle and moderate workloads. However, both technologies exhibit similar power usage under workloads characterized by intensive CPU and memory utilization. These findings are corroborated by Lindström (2022) and Scheepers (2014), whose comparative analyses indicated that containers provide superior memory throughput, enhanced disk I/O performance, and lower operational overhead. In contrast, virtual machines provided stronger isolation mechanisms and more balanced resource allocation.

Several studies employing micro-benchmark analyses have further reinforced the performance advantages of containerization. Potdar et al. (2020) and Preeth et al. (2015) reported that Docker containers achieve near-native or superior performance relative to VMs across standard benchmarks for CPU, memory, and file system operations. Extending beyond synthetic benchmarks, Shirinbab et al. (2020) and Berggren and Karlsson (2022) examined application-level workloads, including database systems and API services. Their findings indicate that while containers generally achieve higher throughput with lower CPU overhead, this performance benefit can be constrained by network I/O limitations in certain deployment configurations.

From the dual perspectives of energy-efficiency and green computing, Anusooya and Vijayakumar (2021) and Morabito et al. (2015b) highlighted that containerized environments consume significantly less energy than VMs when running multiple workloads. These findings highlight the role of containers in enabling more energy-efficient computing infrastructure. However, a notable limitation of this existing body of work is its synthetic benchmarks and its focus on traditional computing resources, such as CPU, memory, storage, and network interfaces.

More recently, Algarni et al. (2024) investigated energy consumption forecasting for Docker containers using time-series modeling. However, that research prioritized predictive accuracy over the analysis of underlying hardware behavior. Although this work represents an advancement in energy prediction methodology, it does not account for the energy profiles of hardware accelerators, such as GPUs, or the distinct power characteristics of specific workload types.

The authors of Atiewi et al. (2018) evaluated the impact of server virtualization on data center energy efficiency using the Power Saver Scheduling Algorithm (PSSA) and green scheduling algorithms within the GreenCloud simulator. The experiments measured performance using three key metrics: makespan, data center load, and server energy consumption. The results showed that virtualized data centers outperformed non-virtualized environments in terms of reducing both energy consumption and overall data center load. However, the virtualized setup introduced a drawback in increased makespan, indicating longer task completion times. The study also emphasized the importance of efficient VM management, highlighting that failures could reduce system throughput and may require VM recovery or migration to maintain performance. It further noted that VM migration, while useful for resource allocation, could negatively affect performance when handling large data transfers.

The authors of Beena et al. (2025) proposed an energy-efficient cloud computing framework that integrated container-based virtualization using Kubernetes with intelligent optimization techniques to reduce power consumption in data centers. The framework leveraged Adaptive Resource Adjustment Algorithm (ARAA) and Automated Resource-Aware Kubernetes Lifecycle Management (ARKLM) to dynamically manage resources, scale workloads, and deactivate idle containers based on real-time demand. It also incorporated optimization methods such as Ant Colony Optimization (ACO), Swarm Intelligence, and Dynamic Voltage and Frequency Scaling (DVFS) to improve workload distribution and energy efficiency. Experimental results showed that DVFS achieved extremely low CPU utilization (0.94%), while ACO provided the fastest execution time, demonstrating a trade-off between energy savings and performance speed.

Khan et al. (2019) investigated energy-efficient resource management in cloud data centers, focusing on how consolidation and migration decisions affected both energy consumption and performance. It highlighted that although resource consolidation reduced energy usage due to underutilized servers, migration introduced significant overhead in terms of energy cost and performance degradation, which was often ignored in existing models. Using real Google workload traces with over 12,000 hosts and one million tasks, the study evaluated different allocation and migration strategies in containerized environments. It proposed an energy-performance-aware allocation method (EPC-FU) combined with a migration strategy (CPER), emphasizing that only long-running containers should be migrated to ensure that migration costs were recovered. The results showed that limiting migrations could sometimes be more efficient than aggressive consolidation, with only a small fraction of containers needing migration while still achieving energy savings.

As summarized in Table 1, the established literature provides comprehensive performance comparisons of virtualization and containerization. These analyses are predominantly centered on traditional resources, such as CPU, memory, disk I/O, and network performance, evaluated under both synthetic and application-level workloads. These studies utilize a diverse range of benchmarking methodologies, spanning from microbenchmarks like 7-Zip and RAMspeed to application-specific deployments such as Apache Cassandra and API hosting. A critical and consistent limitation across this body of research is its general omission of Graphics Processing Units (GPUs). Existing studies are largely GPU-agnostic and thus do not address the escalating centrality of GPU-accelerated computations, particularly for AI and deep learning workloads in contemporary data centers. Notably, prior work lacks analysis of GPU-specific metrics, including power consumption, GPU utilization efficiency, and the consequent environmental and cost implications of running deep learning models on GPUs within containerized environments. Given that GPUs have emerged as the primary drivers of energy consumption in AI infrastructures, this omission represents a significant gap in the literature. This study addresses this identified gap by systematically analyzing GPU power consumption, utilization efficiency, and associated energy cost when executing AI workloads under containerized environments. By employing real, GPU-intensive AI models instead of synthetic, CPU-centric benchmarks, this research provides novel insights into the environmental impact and operational cost of GPU usage.

**Table 1:** Comparison of related work on virtualization and container energy and performance studies.

Study	Technology	Benchmark/workload	Key findings/limitations
Morabito (2015a)	KVM, Xen, Docker, LXC	Sysbench, Iperf, Linux sleep	Containers generally consume less power than VMs in most cases; CPU and memory-heavy workloads show similar power consumption; network performance is similar
Tadesse et al. (2018)	Virtual machines, Containers	Various workloads (CPU, network, idle)	Idle VMs consume more energy than idle containers; CPU and power usage increase with the number of VMs; under CPU load, power is similar; VMs consume more power under network load; demonstrates container energy efficiency in most scenarios
Lindström (2022)	Virtual machines, Containers	7-Zip, RAMspeed, PostMark	Similar CPU performance; containers have better memory performance; disk performance is 15–50% higher for containers, depending on the benchmark; containers consume less power under load, same power when idle; demonstrates containers are more resource-efficient than VMs
Scheepers (2014)	Xen (Hypervisor), LXC (Container)	Microbenchmarks/request handling	LXC handled more requests and processed them faster, reducing overhead; Xen excelled in evenly distributing resources; LXC is ideal for small, isolated processes; Xen is better for tasks requiring balanced resource allocation
Potdar et al. (2020)	Docker, Virtual machines	Prime number computation, 7-Zip	Docker containers consistently outperformed VMs; nearly twice as fast for prime number calculation; 8,200 MIPS vs. 2,000 MIPS for 7-Zip; over three times better memory performance and double the disk performance
Preeth et al. (2015)	Docker	File system performance (Bonnie++)	Docker container performance is nearly identical to the host OS; experiments with 40MB data; demonstrates Docker delivers near-native performance
Shirinbab et al. (2020)	VMware, Docker	Apache Cassandra database (TPS, CPU utilization)	Docker had lower overhead and higher resource efficiency; VMs suffered reduced performance due to operational overhead; multiple instances reduced read performance but increased write performance for both VMware and Docker
Berggren (and Karlsson 2022)	Hyper-V, Docker	API hosting (load tests: hardware utilization, response time, throughput)	Docker may face network bottlenecks, limiting CPU performance; Hyper-V advantages for HTTP-based applications; tests used simulated traffic; Docker is suitable for CPU-intensive workloads with orchestration; VMs are better when CPU speed optimization is less critical
Anusooya (and Vijayakumar 2021)	Virtual machines, Containers	Network load, DVFS modeling, WSM and Trigger-WSM load balancing algorithms	VMs consume significantly more power than containers under network load; a single VM with one application uses more power than four containers each running one application; study demonstrates containers' energy efficiency for green computing
Morabito et al. (2015b)	Host, VMs, Docker, LXC	Y-cruncher, CPU/memory/disk/network benchmarks	Host system fastest, followed by containers, then VMs; CPU and memory differences minimal; VM disk performance 35–50% slower than containers
Algarni et al. (2024)	Docker	Cloud datacenter workloads, multiple applications (time-series energy prediction)	Predicted container energy consumption using AR, ARIMA, and ETS models; ETS has the lowest MAPE for some containers, ARIMA is best for others; highlights the importance of selecting an appropriate time-series model for accurate power prediction
Atiewi et al. (2018)	Virtualization, Non-Virtualization	Simulated cloud data center workloads with VM-based tasks	Virtualization reduces energy consumption and data center load, but increases makespan (longer execution time). VM failures require migration/recovery, which may degrade performance under heavy data transfer
Beena et al. (2025)	Kubernetes-based container	Cloud data center workloads	The framework significantly reduced energy consumption, with DVFS achieving 0.94% CPU usage and ACO providing the fastest execution time. However, it shows a trade-off between energy efficiency and execution speed, requiring balance between performance and energy savings
Khan et al. (2019)	Containerization	Google cluster workload dataset	Migration decisions significantly impact energy and performance; limiting migrations (especially to long-running containers) improved efficiency, with only 1.9% of containers migrated and 61.89% recovering migration cost. However, migration overhead, workload heterogeneity, and runtime estimation remain major challenges, and the model lacks real-world deployment validation.

## 3 Methodology

**Figure 2:**
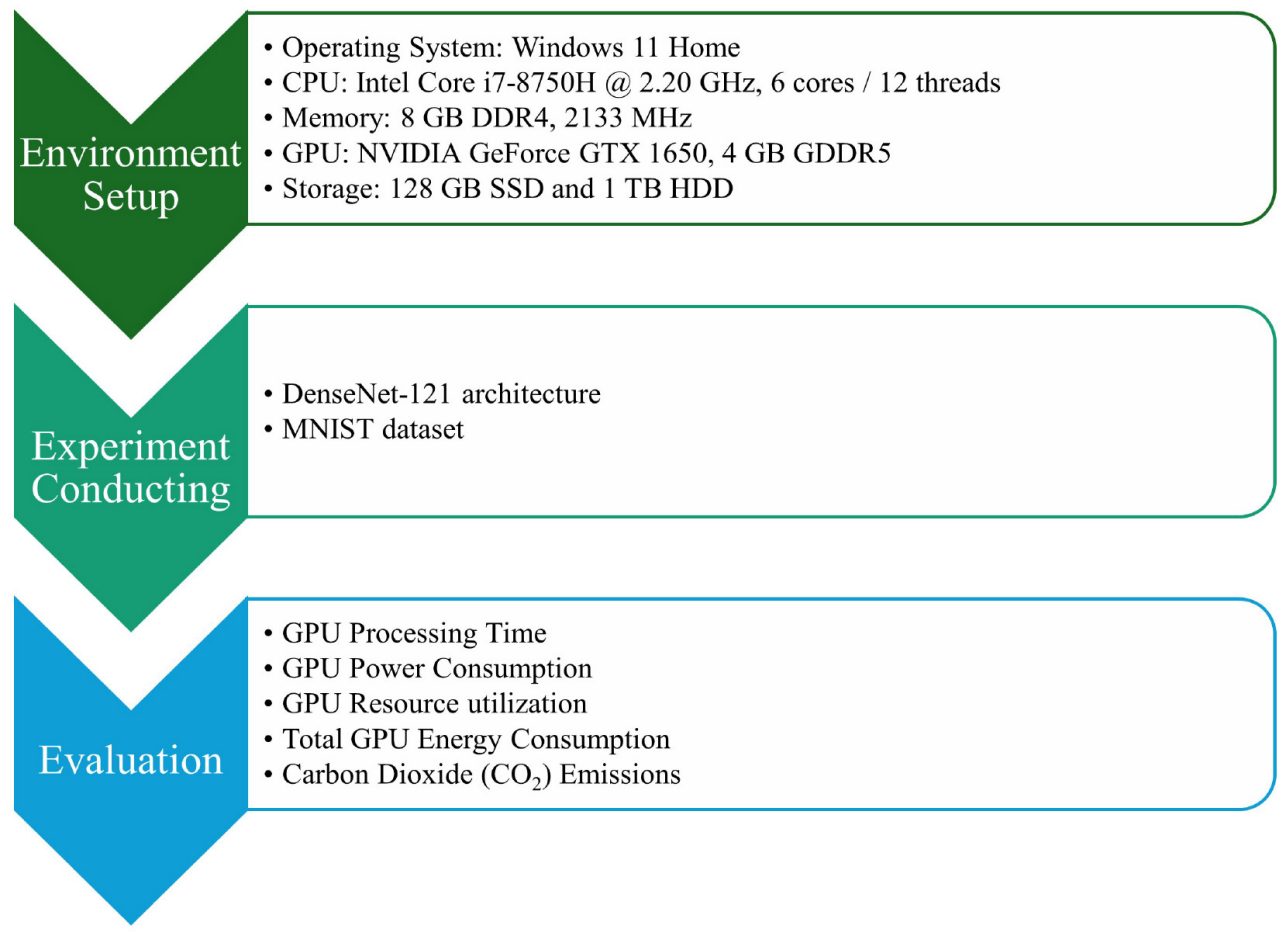
The structure of the research approach.

This section outlines the research methodology employed to address the objectives of this study. The experimental workflow is structured into three sequential phases: environment setup, experiment execution, and performance evaluation, as illustrated in Figure 2. The methodology is designed to ensure fairness, transparency, and reproducibility in the comparative analysis of container-based and virtual machine-based execution environments for deep learning workloads.

### 3.1 Environment setup

To facilitate a controlled comparison, two distinct execution environments were established on a single host machine: a Docker container and a VirtualBox VM. Both environments were configured to execute an identical deep learning workload, using an identical model code, dataset, and training parameters in order to ensure a fair and reproducible comparison between containerization and virtualization technologies. The experimental evaluation focused on performance, power consumption, resource utilization, and environmental impact, with particular focus on GPU usage during AI model training. The host system operated on Windows 11 Home, while Ubuntu 22.10 was used as the guest operating system for both experimental environments. This configuration ensured that differences in observed performance and energy consumption could be attributed to the execution environment rather than operating system discrepancies. The detailed hardware specifications of the host system used in all experiments are listed below:

- Operating System: Windows 11 Home
- CPU: Intel Core i7-8750H @ 2.20 GHz, 6 cores/12 threads
- Memory: 8 GB DDR4, 2,133 MHz
- GPU: NVIDIA GeForce GTX 1650, 4 GB GDDR5, 128-bit bus width
- Storage: 128 GB SSD and 1 TB HDD

For the container-based experiments, Docker was used to create an isolated execution environment. The Docker image was built using the official python: 3.8-slim-buster base image. Essential system-level dependencies, including build-essential tools, Python development headers, and Python package management utilities were installed within the container. All Python dependencies were explicitly defined, including torch and torchvision, to ensure consistency and reproducibility.

A corresponding VirtualBox VM was created and configured with a base memory allocation of 2,048 MB and 16 MB of video memory. The VMSVGA graphic controller was selected with 3D acceleration enabled to support graphical and computational workloads. To ensure experimental consistency, the same deep learning framework, Python version, and software dependencies used in the Docker environment were installed within the VM environments. GPU accessibility and overall system readiness were verified using the same monitoring and validation procedures applied in the container-based setup.

Before executing the experiments, GPU availability and operational status were verified using the nvidia-smi utility, ensuring that the NVIDIA GeForce GTX 1650 GPU was correctly recognized and accessible within the execution environment. The experiments were conducted using NVIDIA driver version 528.49, along with CUDA 11.7 and cuDNN 8.5. The same GPU hardware, driver configuration, CUDA environment, deep learning framework, and software dependencies were maintained consistently across both the Docker container and VirtualBox virtual machine environments to ensure a fair and reproducible comparison. All GPU power consumption and utilization metrics were collected at the host level using NVIDIA driver telemetry tools.

### 3.2 Experiment conducting

The experiments were designed to evaluate the performance and energy-related behavior of virtualization and containerization technologies when executing a representative deep learning workload for computer vision applications. A convolutional neural network (CNN) based on a DenseNet-121 architecture was implemented using the PyTorch deep learning framework (version 2.0.0, Meta AI, Menlo Park, United States) and trained on the MNIST dataset for handwritten digit classification.

The MNIST dataset comprises a training partition of 60,000 images and a test partition of 10,000 images, with each instance represented as a 28 × 28 grayscale image with pixel intensity values ranging from 0 to 255. As a standard preprocessing step, all images were converted to PyTorch tensors and normalized using a mean of 0.1307 and a standard deviation of 0.3081. The MNIST dataset is widely used as a benchmark dataset for image classification tasks, enabling reproducible comparison across experimental studies.

**Figure 3:**
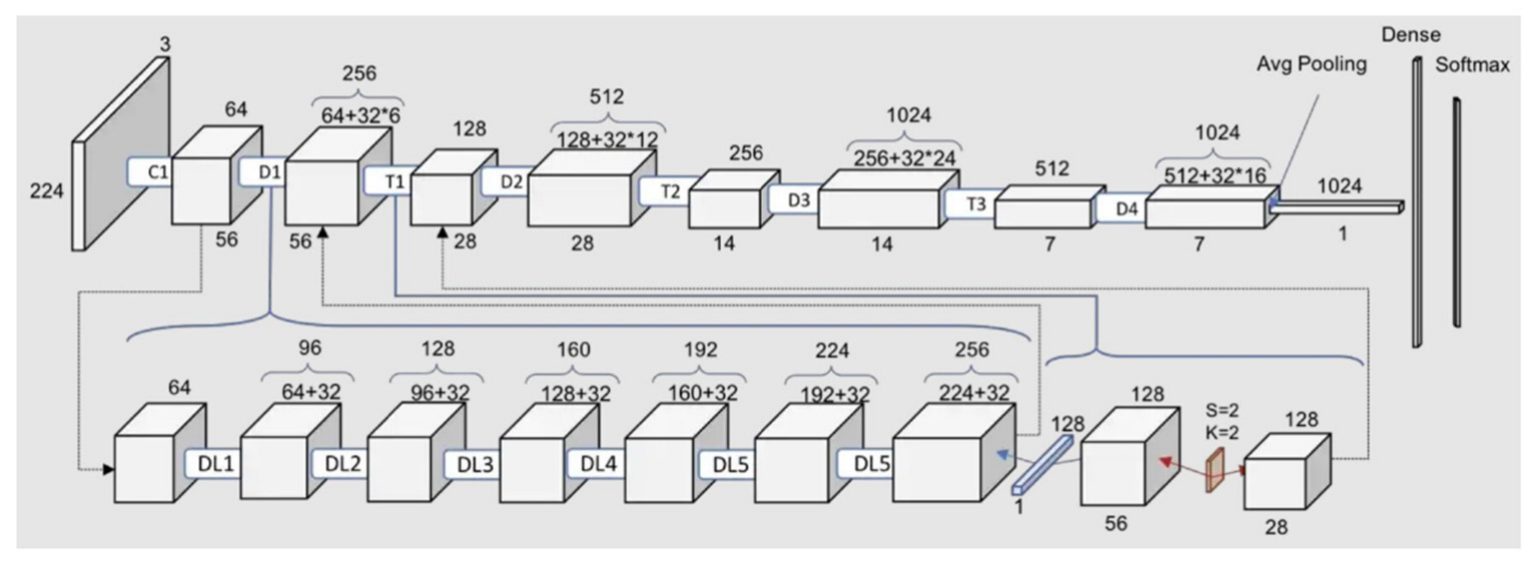
The structure of the DenseNet-based model used in this study (Ruiz, 2018).

The implemented model follows a DenseNet-style convolutional architecture, which is characterized by dense feature propagation between layers. The network comprises multiple convolutional layers with ReLU activation functions and max-pooling operations. Feature channels are progressively increased throughout the network, followed by an adaptive average pooling layer and a fully connected classification layer with ten output neurons corresponding to the digit classes Ruiz (2018). The architecture of the DenseNet-based model used in this study is illustrated in Figure 3.

To maintain the integrity of the comparative analysis, the training configuration was mirrored across both execution environments. The optimization process utilized the Adaptive Moment Estimation (Adam) optimizer, paired with a cross-entropy loss function. Training was conducted for 100 epochs with a batch size of 180. All experiments were executed on the same NVIDIA GeForce GTX 1650 GPU, equipped with 4 GB of GDDR5 memory and a 128-bit memory bus. During training, loss values were periodically logged to monitor learning progression, execution stability, and potential performance fluctuations across the Docker container and VirtualBox VM environments.

### 3.3 Evaluation measures

Following the experimental execution, the results were analyzed to evaluate and compare the GPU efficiency of the Docker container and VirtualBox VM environments. The evaluation was based on primary performance and efficiency metrics, defined as follows:

- GPU Processing Time (seconds): This metric quantifies the total duration required for the GPU to complete the AI model training task within each environment. It serves as a direct measure of computational throughput and processing efficiency, with shorter times indicating higher performance.
- GPU Power Consumption (Watt): This metric captures the instantaneous electrical power, measured in watts (W), consumed by the GPU during workload execution. GPU power data were collected as time-series measurements throughout the training process in both environments. The mean and standard deviation of GPU power consumption were computed to characterize overall energy behavior and variability. GPU power consumption measurement was sampled at 1-s intervals throughout the full training process. Lower average GPU power consumption indicates more efficient utilization of the GPU during AI model training.
- GPU Resource utilization (%): This metric indicates the percentage of the GPU's computational capacity actively engaged during the execution of AI tasks. GPU utilization was sampled continuously during the experiments to assess how effectively each environment leverages the GPU for the given workload. Lower utilization values indicate more efficient workload scheduling and reduced idle GPU time.
- Total GPU Energy Consumption (kWh): This metric captures the cumulative electrical energy required to complete the training workload in each environment. It was calculated by integrating the instantaneous power measurements over time using the formulation shown in Equation 1:

$$\begin{array}{l} E=\overset{n}{\underset{i=1}{\sum }}\left(P_{i}\times \Delta t_{i}\right)/3,600,000 \end{array}$$ (1)

where *P*_*i*_ represents the power consumption in watts at time interval *i*, and Δ*t*_*i*_ denotes the corresponding time interval in seconds. The resulting energy consumption is expressed in kilowatt-hours (kWh).

- Carbon Dioxide (CO_2_) Emissions (kg): To estimate the environmental impact associated with each execution environment, carbon dioxide emissions were calculated based on the total energy consumed. A global average carbon intensity factor of 0.4 kg CO_2_e/kWh was applied (FAIST Group, 2025), as shown in Equation 2:

$$\begin{array}{l} CO_{2}=E\times 0.4. \end{array}$$ (2)

This metric provides an approximate measure of greenhouse gas emissions attributable to running the AI workloads in Docker containers and VirtualBox VM environments.

- Statistical and Time-Series Analysis:

The collected GPU power consumption and utilization measurements were analyzed using statistical and time-series techniques to evaluate the efficiency differences between Docker container and VM. First, block averaging was applied to the raw measurements by grouping consecutive samples into fixed-size windows (60 and 120 samples) and computing their mean values. This approach was used to reduce short-term fluctuations and mitigate the effect of temporal dependence in the data. Descriptive statistics were then computed to summarize the average power consumption and GPU utilization for each virtualization environment. To assess whether the observed differences were statistically significant, independent two-sample t-tests were conducted on the block-averaged data for both power consumption and GPU utilization. In addition, time-series analysis was performed using the ARIMA with configuration ARIMA(1,0,1) to capture temporal dependencies and characterize the dynamic behavior of the power and utilization measurements.

## 4 Results and discussion

The experimental evaluation presents a comprehensive comparative analysis of VirtualBox VM virtualization and Docker-based containerization. The comparison is structured around four key dimensions: system power consumption, GPU resource utilization, total energy usage, and associated carbon dioxide (CO2) emissions. The analysis is based on time-series data collected during the execution of an identical deep learning workload under both environments, ensuring a controlled and equitable basis for comparison. Each epoch required approximately 5 min, resulting in a total training duration of approximately 8 h and 20 min under the tested conditions.

**Figure 4:**
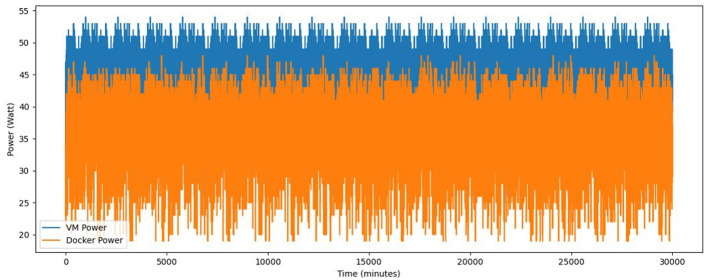
GPU power consumption (W) over time for VirtualBox VM and Docker container.

**Figure 5:**
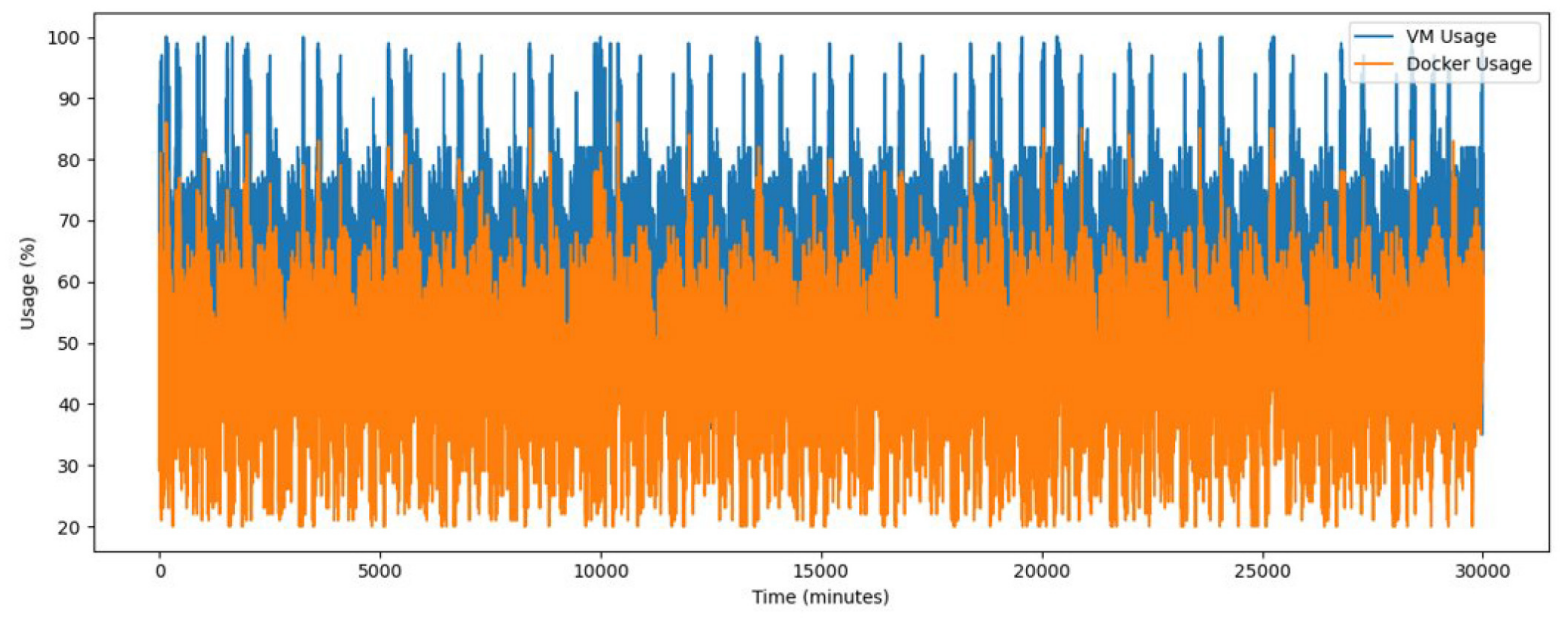
GPU usage (%) over time for VirtualBox VM and Docker container.

The time-series analysis of power consumption (Figure 4) reveals a clear and persistent separation between the two execution environments over the entire execution duration. Power consumption in the VirtualBox VM remains systematically higher, with values fluctuating around a mean of approximately 45.11 W, whereas the Docker container exhibits a substantially lower mean power consumption of approximately 35.37 W. Although both environments display short-term fluctuations corresponding to workload dynamics, the VM configuration demonstrated more pronounced power peaks and a consistently elevated baseline. This pattern can be attributed to the inherent overhead of full hardware virtualization, which requires the execution of a complete guest operating system and hypervisor management processes. Such overheads are largely eliminated in container-based deployments, which rely on shared kernel mechanisms and lightweight isolation. A comparable pattern is evident in the GPU resource utilization time-series shown in Figure 5. Utilization in the VirtualBox VM remains consistently higher, with an average GPU usage of approximately 66.80%, compared with 47.21% observed in the Docker container environment. The elevated utilization and more pronounced usage spikes in the VM environment indicate less efficient resource allocation and increased contention, likely resulting from hypervisor-level scheduling and abstraction overhead. In contrast, Docker exhibits smoother utilization profiles with reduced variance, highlighting the lightweight execution model of containerization and its closer integration with the host operating system.

**Figure 6:**
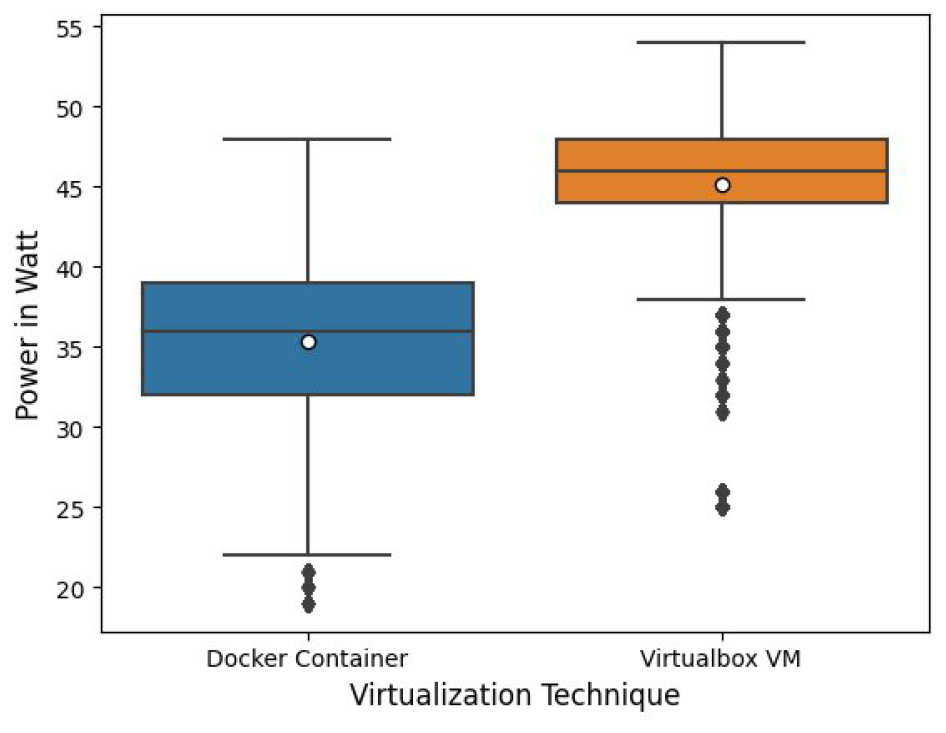
GPU power consumption distribution for VirtualBox VM and Docker container during AI model training in (W).

**Figure 7:**
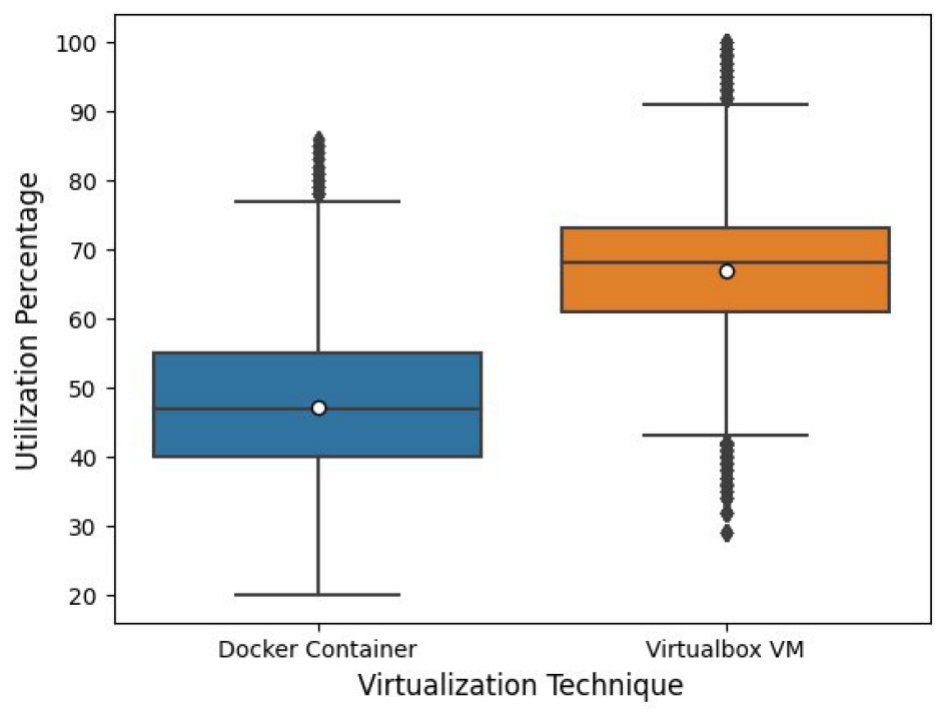
GPU usage distribution for VirtualBox VM and Docker container during AI model training in (%).

The box plot analyses for power consumption and resource usage (Figures 6, 7) further substantiate the initial time-series observations. The median values for both electrical demand and GPU utilization are substantially lower in the Docker environment. Furthermore, the interquartile ranges for the containerized workloads are notably narrower, indicating a more stable and predictable performance. In contrast, the VirtualBox VM box plots exhibit broader dispersion and elevated upper whiskers, confirming the frequent occurrence of high-consumption events. These distributional characteristics demonstrate that the performance differences between the two environments are systematic and persistent, rather than being confined to average values alone.

Quantitative analysis of total energy consumption indicates that the VM environment consumed approximately 0.0125 kWh during the experimental period, whereas the Docker container consumed about 0.0098 kWh. This represents a 21.6% reduction in energy consumption attributable to the use of containerization. Given that cumulative energy is a function of power integrated over time, these findings are fully consistent with the observed power consumption profiles, further validating the efficiency advantage of containerization.

**Figure 8:**
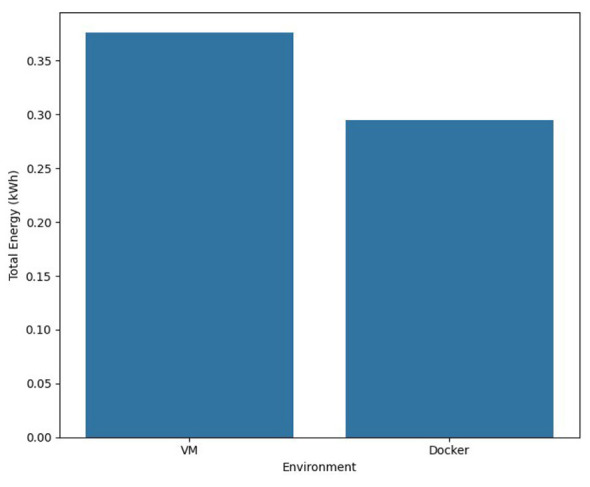
Comparison of total energy consumption (kWh) between VirtualBox VM and Docker environments.

**Figure 9:**
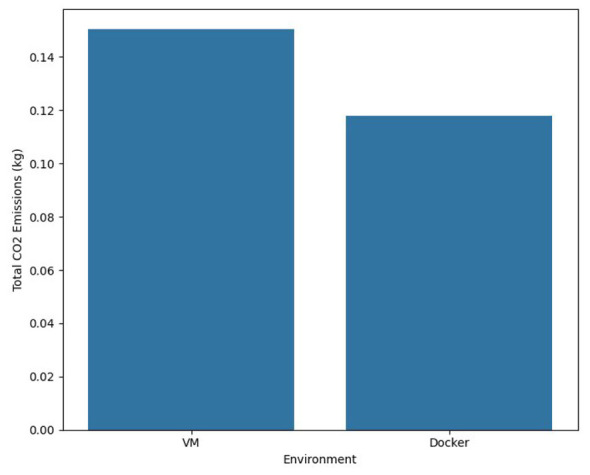
Comparison of total CO_2_ emissions (kg) between VirtualBox VM and Docker environments.

Applying a global average carbon intensity factor of 0.4 kg *CO*_2_e/kWh, estimated carbon emissions were approximately 0.0050 kg *CO*_2_ for the VM and 0.0039 kg *CO*_2_ for the Docker container. The bar charts illustrating total energy consumption and associated *CO*_2_ emissions (Figures 8, 9) clearly demonstrate this disparity, with Docker consistently exhibiting a lower environmental footprint.

The descriptive statistics presented in Table 2 provide an initial comparison between VirtualBox VM and Docker container in terms of GPU power consumption and utilization. Across all measured metrics, Docker consistently demonstrates lower average power consumption (35.37 W compared to 45.11 W for VM) and reduced GPU utilization (47.21% compared to 66.80%). Despite this reduction in resource usage, both environments exhibit comparable levels of variability, as indicated by similar standard deviations in power and GPU utilization. Additionally, Docker also shows lower peak values for both power consumption and GPU usage, suggesting improved efficiency not only on average but also under maximum workload conditions.

**Table 2:** Descriptive statistics of power and GPU usage.

Metric	Virtualbox VM	Docker
Mean power (W)	45.11	35.37
Std. dev power (W)	4.63	5.00
Mean GPU usage (%)	66.80	47.21
Std. dev GPU usage (%)	10.59	10.91
Maximum power (W)	54	48
Maximum GPU usage (%)	100	86

To assess whether these observed differences are statistically significant, independent two-sample t-tests were conducted, with results summarized in Table 3. The findings reveal extremely large t-statistics for both power consumption and GPU utilization under both block sizes (60 and 120 samples), accompanied by near-zero p-values. This confirms that the performance differences between VirtualBox and Docker are statistically significant. Future studies incorporating multiple independent runs may further strengthen the generalizability of these findings. Furthermore, increasing the block size from 60 to 120 samples results in a slight reduction in t-statistic values; however, the significance level remains unchanged, indicating that the observed differences are robust to the choice of aggregation window.

**Table 3:** Comparison of statistical results under different block sizes (60 vs. 120 samples).

Measure	60 samples	120 samples	Interpretation
Power T-statistic	80.62	68.14	Strong and consistent difference; slightly reduced with larger blocks
Power *P*-value	0.0	1.34 × 10^−254^	Highly significant in both cases (p ≪ 0.05)
GPU usage T-statistic	52.84	45.47	Large and stable difference between VM and Docker
GPU usage *P*-value	1.93 × 10^−291^	2.09 × 10^−179^	Extremely significant across both block sizes
VM AR(1) (power)	0.9292	0.9292	Strong temporal dependency; stable across aggregation levels
Docker AR(1) (power)	0.9461	0.9461	Stronger persistence in Docker power time series
VM AR(1) (usage)	0.9318	0.9318	Consistent temporal dependency in VM usage behavior
Docker AR(1) (usage)	0.9568	0.9568	Strong temporal persistence in Docker usage series

In addition to statistical comparison, temporal behavior was analyzed using the ARIMA. The AR(1) coefficients for both environments are consistently high across power and usage metrics, ranging from approximately 0.93 to 0.96. This indicates strong first-order temporal dependency in both VirtualBox and Docker time series, meaning that current measurements are highly influenced by previous values. Notably, Docker exhibits slightly higher AR coefficients, suggesting stronger persistence in its temporal behavior.

Overall, the combination of descriptive statistics, hypothesis testing, and time-series modeling provides a comprehensive evaluation of system behavior. The results consistently demonstrate that Docker containers achieve lower power consumption and reduced GPU utilization compared to VirtualBox, while maintaining stable and predictable temporal dynamics across both workloads and sampling configurations.

Based on the experimental analysis and key findings, the following recommendations are proposed:

1. Adoption of Containerization: Based on the results obtained in this study, containerization demonstrated lower energy consumption and GPU utilization compared to VirtualBox within the evaluated experimental setup. Therefore, in similar controlled environments, containerization may be considered as a more efficient alternative to full virtualization for the tested AI workload.
2. Use of Docker Containers for AI Model Training: The experimental results indicate that, within the tested configuration, Docker containers exhibited lower GPU utilization and reduced power consumption compared to a VirtualBox virtual machine. Accordingly, Docker-based execution may be preferred for AI model training under comparable hardware, software, and workload conditions.
3. Continuous Monitoring and Performance Evaluation: Continuous monitoring of resource utilization and energy consumption can be beneficial when executing AI workloads in virtualized or containerized environments. Such monitoring may help identify optimization opportunities in similar experimental or operational setups.

## 5 Conclusion

This study investigated the environmental and operational impact of virtualization and containerization technologies for GPU-based AI model training within a controlled experimental setup. The work focuses on hardware utilization and energy consumption differences between Docker containers and a VirtualBox VM, addressing a gap in existing green computing studies that have largely emphasized CPU- and memory-centric workloads.

The experimental methodology involved training a DenseNet-121 model on the MNIST dataset for 100 epochs with a batch size of 180 on a single hardware configuration. Each epoch required approximately 5 min, resulting in a total training time of 8 h and 20 min under the tested conditions.

The experimental results demonstrate that Docker consistently outperforms the VirtualBox VM across all evaluated metrics. Specifically, Docker exhibited lower average and peak power consumption, reduced GPU utilization, and more stable resource usage throughout the training process. These efficiency gains translated into approximately 21.6% reduction in total energy consumption and correspondingly lower *CO*_2_ emissions compared with the VM environment. Statistical significance testing further indicated that the observed differences in power consumption and GPU usage were significant, substantiating the robustness and reliability of the findings within the experimental scope of this work.

From a systems perspective, Docker containers demonstrated more efficient GPU allocation and lower power demand, indicating a more energy-efficient execution environment for AI model training. In contrast, the VirtualBox VM consistently showed higher GPU utilization and elevated power consumption, reflecting the additional computational and management overhead associated with full virtualization.

From an environmental perspective, Docker's lower energy requirements directly correspond to a reduced carbon footprint and lower operational power costs. These findings align with the study's objective of evaluating the environmental implications of virtualization technologies and highlight containerization as a promising approach for sustainable AI computing. However, this study represents a proof of concept, conducted under a specific workload, dataset, and hardware configuration. Although the results clearly demonstrate the efficiency advantages of Docker in this controlled setting, broader generalization requires further experimental validation across diverse AI models, datasets, and deployment scenarios.

## Data Availability

The raw data supporting the conclusions of this article will be made available by the authors, without undue reservation.
